# Establishment of an Endoscopy-Guided Minimally Invasive Orthotopic Mouse Model of Colorectal Cancer

**DOI:** 10.3390/cancers12103007

**Published:** 2020-10-16

**Authors:** Chen Chen, Jens Neumann, Florian Kühn, Serene M. L. Lee, Moritz Drefs, Joachim Andrassy, Jens Werner, Alexandr V. Bazhin, Tobias S. Schiergens

**Affiliations:** 1 Department of General, Visceral and Transplantion Surgery, University Hospital, Ludwig-Maximilians-University Munich, Marchioninistr. 15, D-81377 Munich, Germany; Chen.Chen@med.uni-muenchen.de (C.C.); Florian.Kuehn@med.uni-muenchen.de (F.K.); Serene.Lee@med.uni-muenchen.de (S.M.L.L.); Moritz.Drefs@med.uni-muenchen.de (M.D.); Joachim.Andrassy@med.uni-muenchen.de (J.A.); Jens.Werner@med.uni-muenchen.de (J.W.); Alexandr.Bazhin@med.uni-muenchen.de (A.V.B.); 2 Department of Pathology, Ludwig-Maximilians-University Munich, Thalkirchner Str. 36, D-80337 Munich, Germany; Jens.Neumann@med.uni-muenchen.de; 3 German Cancer Consortium (DKTK), Partner Site Munich, Marchioninistr. 15, D-81377 Munich, Germany

**Keywords:** colorectal cancer, orthotopic, mouse model, murine, endoscopy

## Abstract

**Simple Summary:**

Open orthotopic mouse models of colorectal cancer have disadvantages such as the requirement for advanced surgical skills or the trauma caused by laparotomy. To overcome these limitations, this study aimed to evaluate the establishment of an endoscopy-guided minimally invasive model without laparotomy. Different concentrations of the murine CRC cell lines CT26 and MC38 were endoscopically injected into the colorectal wall of BALB/C and C57BL/6J mice, respectively. Consistent tumor growth with the presence of tumor-infiltrating lymphocytes, lympho-vascular invasion, and early spontaneous lymph node, peritoneal, and hepatic metastases were observed. Analysis of the learning curve demonstrated that this model is easy to learn and quick to establish. It enables intra-individual follow-up endoscopies, and features tumors to study mechanisms of metastasis and the interaction with the immune system. The application of specific cell lines and concentrations enables a controlled local tumor growth and metastatic formation within short observation periods.

**Abstract:**

Open orthotopic mouse models of colorectal cancer have disadvantages such as the requirement for advanced surgical skills or the trauma caused by laparotomy. To overcome these drawbacks, this study aimed to evaluate the establishment of a minimally invasive model using murine colonoscopy. CT26 and MC38 CRC cells of different concentrations were injected into BALB/C and C57BL/6J mice, respectively. Follow-up endoscopies were performed to assign an endoscopic score to tumor growth. Gross autopsy, histologic and immuno-histochemical evaluation, and immune scoring were performed. To describe the learning curve of the procedures, a performance score was given. Local tumor growth with colorectal wall infiltration, luminal ulceration, the presence of tumor-infiltrating lymphocytes, lympho-vascular invasion, and early spontaneous lymph node, peritoneal, and hepatic metastases were observed. The tumors showed cytoplasmic immuno-staining for CK20. Compared to the MC38/C57BL/6J model, tumorigenicity and immunogenicity of the CT26/BALB/C model were higher. Tumor volume correlated with the endoscopic score. This endoscopy-guided orthotopic mouse model is easy to learn and quick to establish. It features early metastasis and enables the study of interactions with the immune system. When specific cell concentrations and cell lines are applied, controlled local tumor growth and metastasis can be achieved within short observation periods.

## 1. Introduction

Colorectal cancer (CRC) is a major health problem as it represents the third most commonly diagnosed malignancy and the second leading cause of cancer death worldwide [1]. Factors that influence the patient’s prognosis such as local tumor progression, metastatic spread, and therapy resistance [2] are not sufficiently understood. While in vitro cell culture models such as organoids substantially contribute to improved understanding of the disease [3], these methodological strategies are limited by the absence of pathophysiological microenvironments and the impossibility to study mechanisms of local growth and distant metastasis [4].

Mouse models represent useful in vivo research tools as they allow researchers to gain insights into the mechanisms of CRC progression and metastasis by recapitulating more detailed aspects of human CRC pathophysiology [5,6,7,8]. Among these, implantation models play an important role. Subcutaneous implantation of cancer cells is easy to establish and monitor but does not replicate the original anatomic site [9,10]. Ectopic implantation such as intra-splenic, intra-portal, or intra-hepatic inoculation of cancer cells enables early metastasis but lacks primary tumor growth and represents a rather artificial way of emulating distant spread. Accordingly, orthotopic implantation of cells into the colorectal wall mimics primary tumor growth and spontaneous metastasis apparently in the best way [11]. The most common orthotopic model is created by using open surgical techniques [9]. For this open model, the cecum is exteriorized by laparotomy, and cells are injected into the colorectal wall. Drawbacks of this model include the requirement for advanced surgical skills, the trauma caused by laparotomy, and the impossibility to follow-up on tumor growth or take tumor specimens without sacrificing the animal. Endoscopic implantation may be an attractive alternative to overcome these drawbacks.

Therefore, this study aimed to establish an endoscopy-guided minimally invasive orthotopic mouse model of CRC without laparotomy and to analyze the learning curve as well as the tumor take rate of primary tumor growth and distant spread.

## 2. Results

### 2.1. Learning Curve for Successful Cell Injection

**Figure 1:**
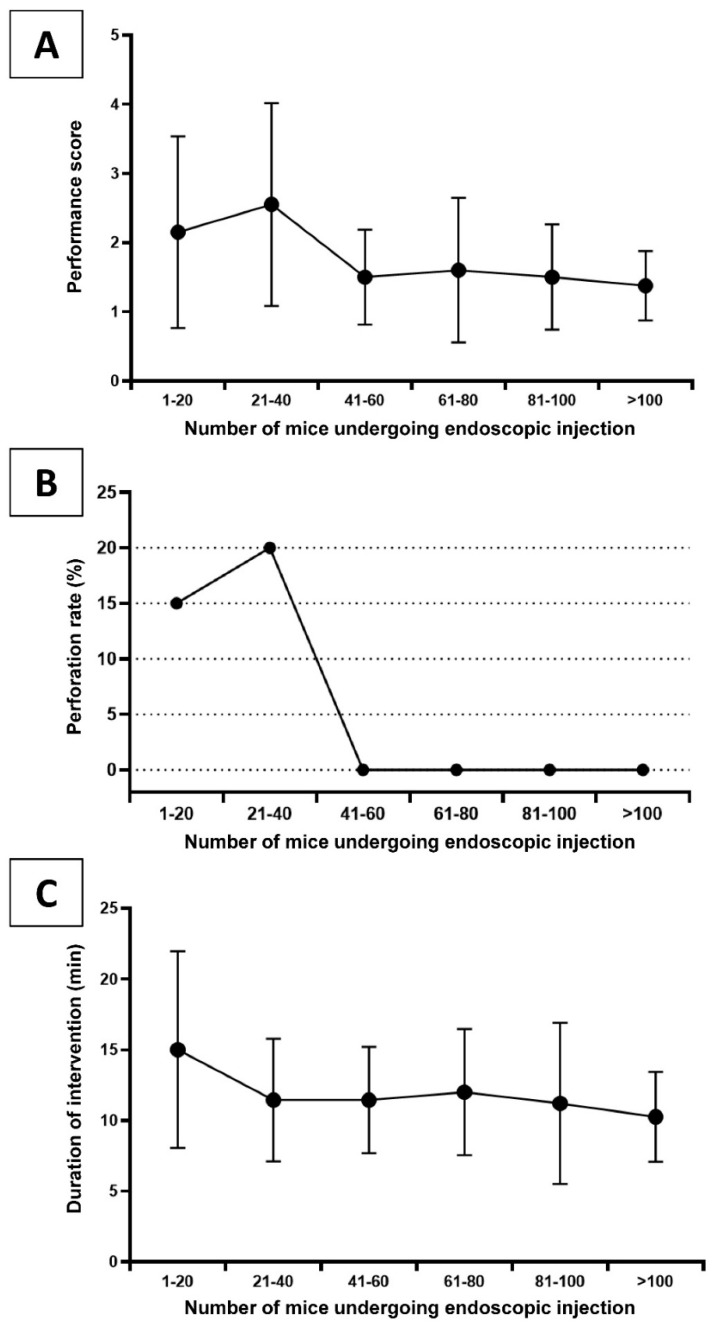
Learning curve during the establishment of the endoscopy-guided tumor cell implantation model showing (**A**) the mean performance score (number of injection attempts with a maximum of 4 attempts, 5 = failure defined as colon perforation or no successful lifting sign), (**B**) the perforation rate, and (**C**) the mean duration of an intervention for mice in chronological subgroups of 20 animals.

Performance score, perforation rate, and the duration of the procedures depending on the number of interventions carried out are shown in Figure 1 Successful injection was achieved in 94% of the interventions with 71% of these attained within the first two attempts (performance score 1 and 2). After 40 procedures, the proportion of score 1 procedures significantly increased (64% vs. 35%, *p* = 0.003). A trend of increased score 1 and 2 procedures was also observed (88% vs. 65%, *p* = 0.060). After 40 interventions, the long-term performance score mean was approximately 1.5 and successful injection was accomplished in 100% of the procedures. The overall perforation rate was 6% with no further adverse events occurring after 40 interventions. The mean duration of the procedure was 12.0 min with a mean of 15.1 min for the first 20 interventions. After that, the duration was significantly reduced to 10.2 min (*p* < 0.003).

### 2.2. Local Tumor Growth

**Figure 2:**
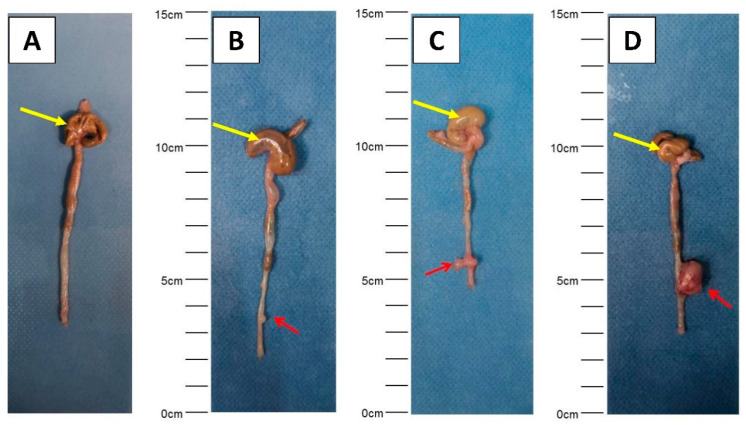
Representative images showing various sizes of primary tumor detected during gross examination ((**A**) negative control, (**B**–**D**) different sizes of primary tumors). Primary tumors are indicated by red arrows (cecum: yellow arrows).

The rates of primary tumor growth including tumor volumes as well as mortality within the observation period are shown in Table 1 and Table 2 for the CT26/BALB/C and the MC38/C57BL/6J models, respectively. Representative images from gross examination are shown in Figure 2.

**Figure 3:**
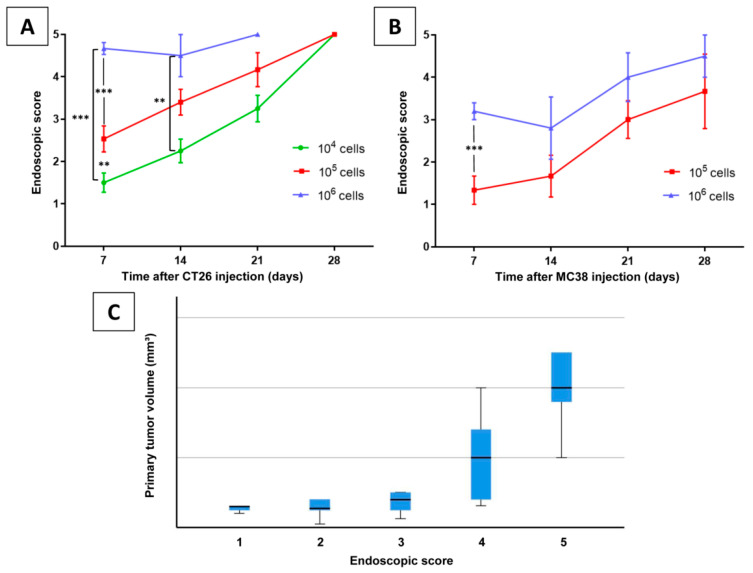
Endoscopic score assigned during follow-up endoscopies on (**A**) BALB/C and (**B**) C57BL/6J mice with varying number of implanted CT26 or MC38 cells, respectively, over an observation period. Significant differences are marked, *p* values represent Bonferroni-corrected values (** *p* < 0.01, *** *p* < 0.001). (**C**) Box plots of primary tumor volume categorized by endoscopic score.

The endoscopic scores during follow-up endoscopies for various numbers of cells over the observation period are depicted in Figure 3A,B. *p* values of comparisons were Bonferroni-corrected. There was no macroscopic or microscopic tumor growth in the negative controls. No differences in tumor growth rates between male and female animals were observed. Compared to the MC38/C57BL/6J model, tumorigenicity of the CT26/BALB/C model was overall higher. In C57BL/6J mice injected with 10^4^ cells as well as mice observed for seven days regardless of the injected cell number, no tumor growth was detected. In this model, the tumor take rate was not improved by increasing the injected cell number from 10^5^ to 10^6^, however, tumor volumes (Table 2) and endoscopic scores tended to be higher (Figure 3B). CT26/BALB/C mice showed high local tumor take rates for all groups with various injected cell numbers and observation periods. After injecting 10^6^ cells, seven animals died or had to be sacrificed due to a high tumor burden according to the score sheet protocols. In this group, the median survival was 18 days (range: 16–19 days). The highest tumor volumes were generated when 10^5^ or 10^6^ cells were injected, resulting in mean volumes of 31 ± 20 (10^5^ cells, 21 days), 23 ± 2 (10^5^ cells, 28 days), 44 ± 45 (10^6^ cells, 21 days group, median follow-up of 18 days due to mortality), and 28 ± 12 mm^3^ (10^6^ cells, 28 days group, median follow-up of 18 days due to mortality), respectively. For both models, primary tumor volume and endoscopic score showed a significant positive correlation (*p* = 0.010). Higher endoscopic scores of 4 and 5 were especially able to predict increasing tumor volumes (Figure 3C).

### 2.3. Lymph Node and Distant Metastasis

**Figure 4:**
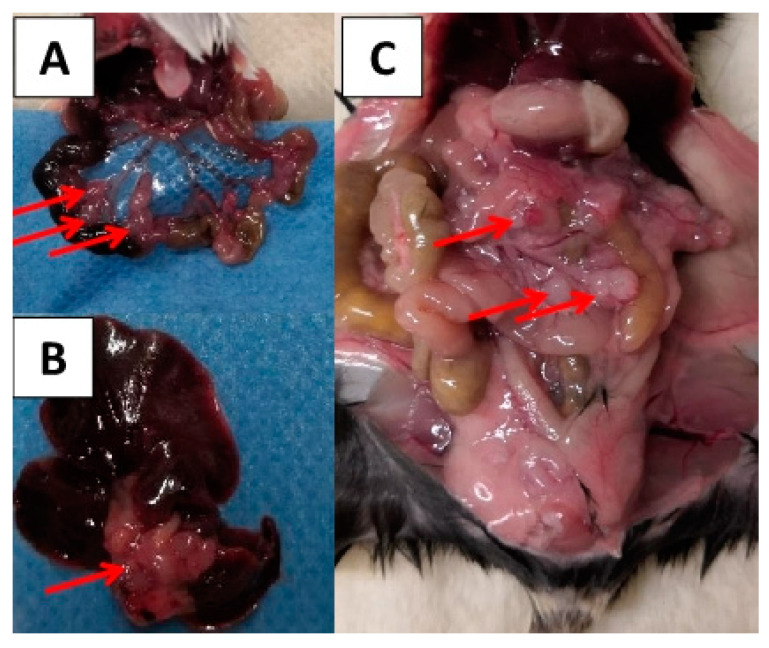
Lymph node metastasis (**A**), liver metastasis (**B**), and peritoneal carcinosis (**C**) detected during gross examination. The metastases are indicated by red arrows.

Regarding lymph node and distant metastasis, the CT26/BALB/C model also revealed a higher tumorigenicity compared to the MC38/C57BL/6J model. No metastatic growth was found in negative controls and animals without primary tumor growth. No differences in rates of distant metastasis between male and female animals were observed. Examples of gross examination are shown in Figure 4.

In MC38/C57BL/6J mice, lymph node metastasis occurred only in three animals after 28 days of observation and when a minimum of 10^5^ cells was applied (Table 2). Peritoneal carcinosis was detected in only one animal. No further distant spread was found in these animals. In the CT26/BALB/C group, lymph node metastases were detected in up to 100% of the animals except for the subgroup with 10^4^ cells and seven-days observation. As distant metastases, peritoneal carcinosis was found in three animals irrespective of cell number or time point. Furthermore, hepatic metastases were found in up to 50% of animals with primary tumor growth (Table 1).

### 2.4. Histology and Immuno-Histochemistry

**Figure 5:**
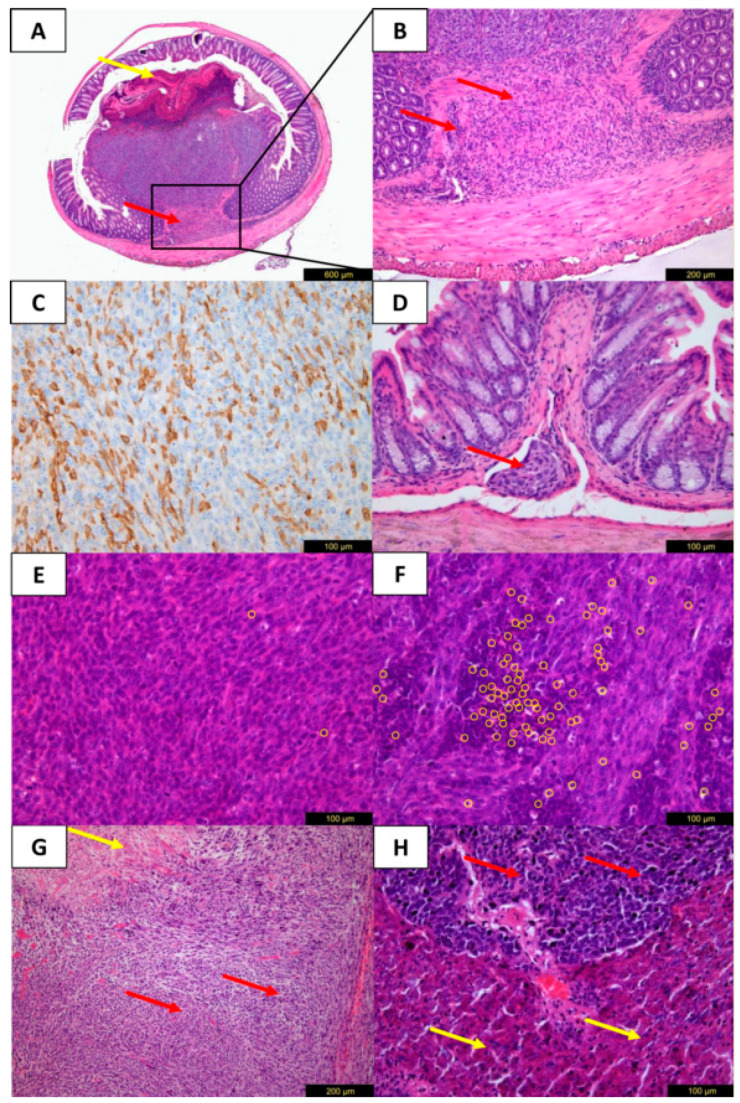
Microscopic tumor evaluation of the CT26/BALB/C model with H&E (**A**,**B**,**D**–**H**) and immuno-histochemical staining for CK20 (**C**). Transverse section of the colon near the injection site with growth of the primary tumor. The malignant tumor with moderate atypia of the nuclei shows luminal ulceration (**A**, yellow arrow) and infiltration of the *muscularis propria* layer (**A**,**B**, red arrows) corresponding to a pT2 category in humans. (**C**) Cytoplasmic positivity of tumor cells for CK20. (**D**) Lympho-vascular invasion (red arrow) corresponding to an L1 category in humans near primary tumor site. (**E**,**F**) Scoring of stromal tumor-infiltrating lymphocytes (yellow circles) with a score of 1 (0–10% stromal TILs, **E**) and a score of 6 (>50% stromal TILs, **F**). (**G**) Mesenteric lymph node metastasis with tumor infiltrates (red arrows) and necrosis (yellow arrows). (**H**) Liver metastasis with tumor infiltrates (red arrows), normal liver tissue (yellow arrows).

Tumors of both models showed a similar microscopic malignant phenotype with a moderate atypia of the nuclei. Representative histologic and immuno-histochemical stainings of the CT26/ BALB/C model are shown in Figure 5. As morphologic characteristics, infiltrating growth into the colorectal wall and lympho-vascular invasion were noticed (Figure 5A,B,D). In addition, luminal ulceration of primary tumors was observed frequently. TILs scoring (Figure 5E,F) revealed a higher mean score for the CT26/BALB/C model compared to the MC38/C57BL/6J group (3.0 ± 1.4 vs. 1.5 ± 0.9; *p* = 0.003). Comparing the different groups of cell numbers or observation periods within one mouse strain, no significant differences in the TILs scores were found. Tumors of both models showed cytoplasmic positivity for CK20 (Figure 5C) with concurrent positive staining of the normal epithelium.

## 3. Discussion

In the present study, a syngeneic endoscopy-guided minimally invasive orthotopic mouse model of CRC was established quickly and easily with a short generation time. It was shown to closely resemble human CRC featuring local tumor growth with colorectal wall infiltration, luminal ulceration, the presence of tumor-infiltrating lymphocytes, lympho-vascular invasion, and lymph node, peritoneal, and hepatic metastases.

Despite improvements of in vitro or ex vivo models such as organoids, in vivo models of CRC are still indispensable tools to improve the understanding of disease progression, metastasis, interaction with the immune system, and drug resistance. Over 95% of animal studies are conducted in mice due to their similarities to humans regarding anatomy, physiology, and genetics [12,13]. Orthotopic mouse models with injection of cancer cells into the colorectal wall are reliable tools to mimic local tumor growth and metastasis [14]. The tumors of these models have similar histology to humans, exhibit metastatic potential, replicate local tumor invasion including lympho-vascular invasion, and allow genetic manipulation [14]. Xenograft models are widely used; however, they have a limitation whereby immune-deficient mice are required and hence the crucial role of the immune system cannot be studied [14]. Syngeneic orthotopic models enable the study of tumor-infiltrating cells such as T cells, B cells, and NK cells. Furthermore, these models are suitable for testing the efficacy of checkpoint blockade therapy [10]. The model presented in our study showed different degrees of TILs with a higher immunogenicity in the CT26/BALB/C group. Together with its other features such as spontaneous metastasis, it could be used to answer important questions regarding immune response and distant spread.

The most amply used model is the open orthotopic model, in which carcinogenic cells or intact tumor tissue are injected into the cecal wall after laparotomy of the animal [9,14]. Inflammatory response caused by laparotomy is a potential bias affecting research on oncoimmunology and studies to determine the suitability of immunotherapy [10,15]. The surgical trauma may significantly affect the immune response and tumorigenicity [16,17].

In 2006, mouse endoscopy was introduced by Becker et al. [18] followed by further investigations and applications of different models with various endoscopy systems. Zigmond et al. [19] presented a successful injection model with implantation of murine and human CRC cell lines. In contrast to our study, the authors did not show distant metastasis. Beyaz et al. [20] injected primary progenitor cells (LGR5-GFP^hi^ *Apc*-null intestinal stem cells, LGR5-GFP^low^ *Apc*-null cells) and were able to show that PPAR-delta activation bestows adenoma-initiating capacity to *Apc*-null progenitors. Roper et al. presented a variety of endoscopy-guided injection CRC models and showed the efficiency of different highly innovative approaches including CRISPR/Cas9-mediated in situ *Apc* editing and transplantation of different organoids including patient-derived primary CRC organoids [21]. The authors demonstrated consistent tumor growth and metastasis. In addition, the engrafted organoids could be visualized in vivo by fluorescence colonoscopy [21]. In the present study, the establishment process of the current model is evaluated in detail and learning curves are investigated. The easy syngeneic setting using varying concentrations of two common murine CRC cell lines with different observation periods and the detailed data on the outcomes in these groups provide a guide for a defined experiment setting depending on the scientific aim. 

The advantages of the current endoscopic model include avoidance of the animal’s laparotomy and the possibility of minimally invasive intra-individual endoscopic follow-up to monitor and score tumor growth. Follow-up endoscopy represents a unique advantage of the current model especially since no perforation or mortality occurs during this process. Furthermore, it has been shown that endoscopic scores of 4 and 5 predict a higher primary tumor volume found during autopsy. This minimally invasive technique can not only be used for monitoring local tumor growth but can also be used to perform consecutive biopsies from the same animal through the working channel of the endoscope. Furthermore, given the increasing knowledge about differences between left-sided and right-sided CRC [22], this technique addresses the need for left-sided CRC models [14]. In the endoscopic model, the interaction between tumor cells and the microenvironment takes place in the distal colorectum which could make this model more suitable for research focusing on this part of the colorectum. Injection could even be performed in a more distal position to model rectal cancer aimed at the development of spontaneous lung metastasis.

Overall, more advanced surgical skills are needed for the open model [9], and endoscopy might be faster and easier to learn. Open-cell implantation is considered a challenging procedure with inflammation of the implanted site [23]. For the open model, technical problems and the incidence of postoperative adverse events or mortality are not reported in detail by many authors. In one study, bowel dilatation, obstruction, and ischemia due to extensive adhesions were reported, impairing the experiments by a significant reduction in overall survival [24]. In another study, postoperative adverse events were reported in 20% of the animals including inflammation of the injection site, bowel leakage, and abdominal bleedings [25]. In our study, we show that murine endoscopy can be learned fast by researchers, when aspects of successful human endoscopy such as adequate bowel cleansing and air insufflation are considered. After approximately 40 procedures, a high level of cell injection performance was reached and no perforation occurred in further procedures. In addition, the duration for a safe intervention with cell injection could be reduced after 20 procedures to approximately 10 min per animal including anesthesia. For endoscopists who are interested in in vivo CRC models, the proposed technique should be easy to learn. 

In our study, successful cell implantation was achieved in 94% of the interventions. In comparison, Zhao et al. reported an 80% success rate for implantation [10]. They detected tumors three weeks after implantation of 10^5^ CT26 cells. In open-injection models, the tumor take rate ranges from 60% to 70% [14]. Discussed reasons for these moderate results were incorrect injection (trans-mural, intra-luminal), low viability of tumor cells, and host reaction to the cells [14]. In our study, the CT26/BALB/C model had a high primary tumor take rate of overall 92% with a low perforation rate. In addition, trans-mural injection and perforation were immediately noticed by pneumoperitoneum. For studying local tumor growth, low cell numbers (10^4^) will be sufficient, resulting in increasing primary tumor volumes over time (Table 1).

Regarding spontaneous distant metastasis, we observed early development in up to 50% of mice after 14 days when higher cell numbers of CT26, especially 10^6^, were injected. These animals, however, showed high tumor burden after two weeks with mortality as a consequence. With regard to metastasis development in orthotopic models, the tumor take rate has been reported to be low. In addition, metastasis occurred only after a long observation period. In fact, only few studies using orthotopic injection report the development of distant spread [26]. Bettenworth et al. used a xenograft approach with human HT-29 CRC cells and reported liver and peritoneal metastasis in 29% and 14% of the animals, respectively. When we injected 10^6^ CT26 cells, we were able to achieve metastasis in 25% of the animals. In the current study, it must be noted that metastases were observed in immune-competent animals after a short observation time.

Some technical considerations should be kept in mind to ensure the success of the current model. Based on publications of Becker et al. [18] and Kodani et al. [27], the valuable advice of experts and pioneers in the field of murine colonoscopy, Professor Becker, and Professor Varol as well as personal experience, a checklist with tips for the establishment of this model is presented in Table 3.

This endoscopic model has some limitations. First, costs for the one-time purchase of an endoscopic system compared to the surgical instruments for an open approach are higher. In addition, as an orthotopic model, the observation period is limited by the incidence of bowel obstruction or high tumor burden. Seven animals had to be sacrificed due to a high tumor burden according to the score sheet before the planned end point was reached (Table 1). These mice underwent injection of the highest cell number of the more tumorigenic and biologically aggressive CT26 cell and developed tumor-related bowel obstruction with consecutive ileus. All other animals in this study—especially C57BL/6J mice undergoing MC38 injection—remained in good condition until the end of the observation period. 

In this study, we present an endoscopy-guided orthotopic mouse model of CRC that is easy to learn and that can be established quickly. It takes a short time per procedure, enables intra-individual follow-up endoscopies, and features the presence of tumor-infiltrating lymphocytes, lympho-vascular invasion, and early spontaneous lymph node, peritoneal, and hepatic metastases. We show data proving that controlled local tumor growth and distant metastasis can be achieved within short observation periods when specific cell concentrations and cell lines are applied. Given the tumor growth rates, sizes of tumors, the general good condition of the majority of the animals, and their survival (Table 1 and Table 2), it is very likely that this model can be used to study application of systemic therapies (i.e., chemotherapy) [28,29]. The data collected by this study allow researchers to quickly and easily implement this model for their research and this model can also be adapted to allow tailored experiments depending on their specific research aim (e.g., primary tumor growth, metastasis). 

## 4. Materials and Methods 

This study was approved by the responsible animal care committee (ROB-55.2-2532.Vet_02-17-110). All experiments were performed in compliance with the guidelines for animal protection in Germany and those of the Federation of European Laboratory Animal Science Associations [30]. The preparation of the manuscript was carried out following the ARRIVE guidelines [31]. 

### 4.1. CRC Cell Lines

For syngeneic orthotopic injection into the colorectum of immune-competent BALB/C and C57BL/6 mice, two murine CRC lines, CT26 (American Type Culture Collection; ATCC, Manassas, VA, USA) and MC38 (gift of Professor Wolf’s lab, Gene Center and Department of Biochemistry, LMU) were injected into the respective source mouse strains. CT26 cells were cultured in RPMI-1640 medium (Gibco, Paisley, UK) with 10% fetal bovine serum (FBS, Biowest, Nuaillé, France) and 1% penicillin-streptomycin (pen-strep, Pan-Biotech, Aidenbach, Germany). MC38 was cultured in Dulbecco’s modified Eagle’s medium (Gibco, Paisley, UK) with 10% FBS and 1% pen-strep. Both cell lines were authenticated before the study. For cell implantation, cells in the log phase but still sub-confluent were collected, and a single cell suspension was prepared in Dulbecco’s Buffered Salt Solution (DPBS, Pan-Biotech, Aidenbach, Germany) and kept on ice. The CASY Cell Counter & Analyzer System (OLS OMNI Life Science, Bremen, Germany) was used to evaluate cell viability before implantation, and only aliquots with a viability of at least 90% were injected.

### 4.2. Mice

Male and female (1:1) BALB/c mice and C57BL/6J mice aged 10 to 11 weeks (Charles River, Sulzfeld, Germany) were used. The animals were housed in groups of five in Makrolon^®^ type II cages (Tecniplast, Hohenpeissenberg, Germany) containing low-dust softwood fiber bedding material. Plastic play tunnels and igloos as well as nesting material were provided for animal enrichment. Mice were maintained in a 12-h light/dark cycle, provided with normal pelleted chow food (Ssniff, Soest, Germany) and tap water ad libitum. Animals were randomized into groups (cell concentration, observation period). As no reliable sample size calculation was possible, a subgroup size of four animals was granted by the animal care committee.

### 4.3. Anesthesia and Endoscopy

**Figure 6:**
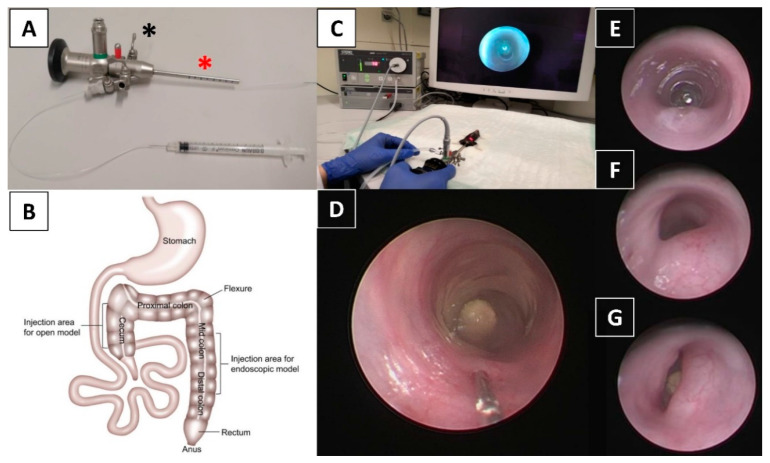
Endoscopy-guided minimally invasive orthotopic mouse model of colorectal cancer using the Mainz Coloview mini-endoscopic system. (**A**) Zero-degree optic (1.9 mm) with a 9 Fr. sheath including a 3 Fr. working channel and a gradation for insertion control (red asterisk). The valve of the Luer lock adapter (black asterisk) is adjusted to create a slow constant air flow. (**B**) Murine anatomy of the colorectum showing the injection sites of open models (injection via laparotomy) [32] and the endoscopic model of the current study. Injection is performed into the lower mid and distal colon in this study. (**C**) Examination and injection with a mouse in dorsal position on a covered heated pad. (**D**) The colonic mucosa is gently penetrated in an approximately 20° to 30° angle using an attached 31 G needle and 50 μL of tumor cell suspension is injected. Representative images showing different endoscopic scores of endoluminal tumor growth: (**E**) score of 3 (~1/4 of the lumen), (**F**) score of 4 (~1/2 of the lumen), (**G**) score of 5 (>1/2 of the lumen).

For anesthesia, medetomidine (0.5 mg/kg, Zoetis, Berlin, Germany), midazolam (5 mg/kg, Ratiopharm, Ulm, Germany), and fentanyl (0.05 mg/kg, Albrecht, Aulendorf, Germany) were administered via intraperitoneal injection. The depth of anesthesia was assessed using toe pinch observing no withdraw reflex (stage of surgical tolerance). Peri-interventionally, mice were placed on a heated pad (Witte & Sutor, Murrhardt, Germany) for the maintenance of homeostasis. For endoscopy and endoscopy-guided tumor cell implantation, the Coloview^®^ system (Storz, Tuttlingen, Germany) with a zero-degree optic (diameter 1.9 mm) was utilized (Figure 6A). The colon was then washed gently with 2 mL of DPBS at 37 °C using a soft pipette (Nerbe plus, Winsen/Luhe, Germany). For endoscopy without cell implantation, a 7 Fr. examination sheath was used. The valve of the Luer lock adapter (Figure 6A, black asterisk) was adjusted to create a slow constant air flow which made the mucosa just become flattened after gentle trans-anal insertion of the endoscope. This enabled a clear 360-degree view without inflating the gastrointestinal tract too severely. The extent of insertion was controlled endoscopically by using gradations on the endoscope (Figure 6A, red asterisk).

### 4.4. Endoscopy-Guided Tumor Cell Implantation

All endoscopic procedures were performed by two investigators, a veterinarian with expertise in small animal care but no expertise in endoscopic techniques (CC) and an attending surgeon (in human medicine) with over 5 years of experience in human endoscopy (TSS). All animals were endoscopically examined to ensure that colonic mucosa was healthy prior to implantation. For tumor cell implantation, a 9 Fr. examination sheath containing a 3 Fr. working channel was applied (Figure 6A,C). After insertion of the endoscope to 30 ± 5 mm from the anal verge into the lower mid and distal colon (Figure 6B), a flexible injection catheter (inside diameter 0.28 mm, outside diameter 0.61 mm, Smiths Medical International, Kent, UK) was introduced through the working channel and the colonic mucosa was gently penetrated in an approximately 20–30° angle (Figure 6D) using an attached 31 G needle with its bevel directed towards the lumen. Subsequently, 50 μL of tumor cell suspension was injected very slowly using the Omnican^®^ F syringe (B. Braun, Melsungen, Germany) attached to the other side of the catheter (Figure 6D; Video S1). A characteristic lifting of the mucosa during injection indicated successful implantation (Video S1). If submucosal introduction of the needle was not successful, up to three further attempts were undertaken. For negative controls, mice were injected with DPBS only or had no endoscopy before follow-up. Three different cell concentrations (10^4^, 10^5^, or 10^6^ cells per 50 µL of injection volume) and four observation periods (7d, 14d, 21d, 28d) were chosen. These experimental groups are shown in Table 1 and Table 2. Only animals with successful implantation indicated by a positive lifting sign and without intra-interventional perforation were entered into these groups. After injection, the animals were assessed daily according to a standardized score sheet to assess post-operative adverse events. The tumorigenesis of mice was endoscopically monitored weekly and tumor growth was recorded according to an endoscopic score [18]: tumor just detectable (1), tumor’s size/diameter 1/8 (2), 1/4 (3), 1/2 (4), or >1/2 (5) of the colorectal lumen. Representative images showing a score of 3, 4, and 5 are shown in Figure 6E–G.

For analysis of the learning curve, performance data from interventions carried out on 116 mice that were chronologically grouped into blocks of 20 mice with the last group (>100) containing 16 animals were analyzed. To describe the learning curve, quality indicators such as success rate of submucosal introduction of the needle and injection into the colorectal wall, perforation, post-interventional mortality, and the duration of the procedure were recorded. Successful submucosal injection was defined as observation of a positive lifting sign of the mucosa indicating no transmural injection and absence of mucosal bleeding. Perforation was defined as endoscopically observed bowel perforation or a macroscopically obvious pneumoperitoneum. A performance score was graded according to the number of attempts needed for successful injection and with a score of 5 defined as no successful injection or perforation. The duration of the procedure was the time from the beginning of anesthesia to removal of the endoscope. 

### 4.5. Autopsy and Tumor Assessment

At the end of the observation period, the animals were sacrificed by cervical dislocation. Autopsy was performed according to the Treuting’s guideline [33]. After sectioning and staining for histological examination, the presence of neoplasm was assessed at the localization of injection, and in mesenteric tissue, the liver, the lung, and any tissue showing abnormalities. The locations, number as well as the size of the tumor and metastasis were recorded. Tumor volumes were calculated as previously reported [34]. 

Hematoxylin and eosin (H&E) staining was performed according to standard protocols. Scoring of tumor-infiltrating lymphocytes (TILs) [35,36] was performed on tumor tissue slides containing the invasive tumor margin by two independent observers (CC, JN). Discrepancies were resolved by a consensus decision. For TILs scoring, only tumor stroma areas were assessed, and the percentages of stromal TILs were classified as 0–10% (1), 11–20% (2), 21–30% (3), 31–40% (4), 41–50% (5), and >50% (6) [36]. Immuno-histochemical staining for cytokeratin (CK) 20 (1:200, Progen Biotec, Heidelberg, Germany) was performed according to standard protocols [37,38].

### 4.6. Statistical Analysis

All data were expressed as mean ± standard deviation or as numbers and percentages. Categorical data were analyzed using either the chi-square test or Fisher’s exact test. Continuous variables were compared using the Student’s t-test and one-way ANOVA with the Bonferroni correction applied for multiple comparisons where indicated. Correlation was assessed using bivariate correlation. *p* values less than 0.05 were considered statistically significant. The data were analyzed using GraphPad Prism (version 8.4.2, GraphPad, San Diego, CA, USA) and SPSS (version 25.0, SPSS, Chicago, IL, USA).

## 5. Conclusions

The endoscopy-guided orthotopic mouse model of colorectal cancer presented in this study is easy to learn and quick to establish. It enables intra-individual follow-up endoscopies, features early metastasis and enables the study of interactions with the immune system. When specific cell concentrations and cell lines are applied, controlled local tumor growth and metastasis can be achieved within short observation periods.

## Figures and Tables

**Table 1:** Outcomes after endoscopy-guided injection of CT26 cells into BALB/C mice: mortality within the observation period, tumor take rate, tumor volume, and the rates of distant metastasis in experimental groups with varying number of cells injected or observation periods.

Number of Injected Cells	Observation Period (d)	Mortality [mice(%)]	Tumor Take Rate [mice(%)]	Tumor Volume (Mean ± SD, mm^3^)	Metastasis Rate [mice(%)] ^a^		
					Mesenteric Lymph Node Metastasis	Peritoneal Carcinosis	Hepatic Metastasis
10^4^	7	0/4(0)	2/4(50)	2.5 ± 0.7	0/2(0)	0/2(0)	0/2(0)
	14	0/4(0)	4/4(100)	2.6 ± 1.5	3/4(75)	0/4(0)	1/4(25)
	21	0/4(0)	4/4(100)	5.8 ± 2.9	1/4(25)	1/4(25)	0/4(0)
	28	0/4(0)	4/4(100)	20.0 ± 1.6	1/4(25)	0/4(0)	0/4(0)
10^5^	7	0/4(0)	4/4(100)	4.8 ± 5.2	2/4(50)	0/4(0)	1/4(25)
	14	0/4(0)	4/4(100)	7.0 ± 7.4	4/4(100)	1/4(25)	2/4(50)
	21	0/4(0)	3/4(75)	31.0 ± 20.7	2/3(67)	0/3(0)	0/3(0)
	28	0/4(0)	3/4(75)	22.7 ± 2.3	1/3(33)	0/3(0)	0/3(0)
10^6^	7	0/4(0)	4/4(100)	31.3 ± 17.7	2/4(50)	0/4(0)	1/4(25)
	14	0/4(0)	4/4(100)	15.8 ± 8.5	2/4(50)	0/4(0)	2/4(50)
	21	3/4(75) ^b^	4/4(100)	44.3 ± 45.2	2/4(50)	0/4(0)	0/4(0)
	28	4/4(100) ^b^	4/4(100)	27.5 ± 11.9	2/4(50)	1/4(25)	1/4(25)

^a^ Mice with metastasis/mice with primary tumor growth in the group. ^b^ These animals died or had to be sacrificed due to a high tumor burden according to the score sheet protocols. The median survival was 18 days (range: 16–19 days).

**Table 2:** Outcomes after endoscopy-guided injection of MC38 cells into C57BL/6J mice: mortality within the observation period, tumor take rate, tumor volume, and the rates of distant metastasis in experimental groups with varying number of cells injected or observation periods.

Number of Injected Cells	Observation Period (d)	Mortality [mice(%)]	Tumor Take Rate [mice(%)]	Tumor Volume (Mean ± SD, mm^3^)	Metastasis Rate [mice(%)] ^a^		
					Mesenteric Lymph Node Metastasis	Peritoneal Carcinosis	Hepatic Metastasis
10^4^	7	0/4(0)	0/4(0)	-	0/0(0)	0/0(0)	0/0(0)
	14	0/4(0)	0/4(0)	-	0/0(0)	0/0(0)	0/0(0)
	21	0/4(0)	0/4(0)	-	0/0(0)	0/0(0)	0/0(0)
	28	0/4(0)	0/4(0)	-	0/0(0)	0/0(0)	0/0(0)
10^5^	7	0/4(0)	0/4(0)	-	0/0(0)	0/0(0)	0/0(0)
	14	0/4(0)	1/4(25)	4.0 ± 0.0	0/1(0)	0/1(0)	0/1(0)
	21	0/4(0)	2/4(50)	2.2 ± 1.3	0/2(0)	0/2(0)	0/2(0)
	28	0/4(0)	3/4(75)	9.7 ± 5.5	2/3(67)	1/3(33)	0/3(0)
10^6^	7	0/4(0)	0/4(0)	-	0/0(0)	0/0(0)	0/0(0)
	14	0/4(0)	2/4(50)	10.0 ± 8.5	0/2(0)	0/2(0)	0/2(0)
	21	0/4(0)	1/4(25)	18.0 ± 0.0	0/1(0)	0/1(0)	0/1(0)
	28	0/4(0)	2/4(50)	14.1 ± 15.5	1/2(50)	0/2(0)	0/2(0)

^a^ Mice with metastasis/mice with primary tumor growth in the group.

**Table 3:** Checklist of steps and tips for successful establishment of the current endoscopy-guided mouse model of colorectal cancer.

**Before Anesthesia**
⬜ Mark gradations on the endoscope sheath to facilitate recording of injection location/tumor position
⬜ Set the light intensity to ~70% and do not change it during the whole procedure to avoid bias
⬜ Set the white balance by pointing the telescope/camera directly at a white object 3–5 cm away
⬜ Set the focus so that objects at a distance of 3–5 mm give a crisp picture
**Before Endoscope Induction**
⬜ Carefully examine the perianal area of the animal to ensure there are no lesions
⬜ Make sure the needle is completely inside the sheath. An exposed needle during endoscope insertion may cause harm to the colon and perforation
⬜ Adjust the valve of the Luer lock adapter until a slow constant flow of air is observed when the needle is submerged in a tube of water
⬜ Rinse the endoscope in warm PBS for lubrication and to avoid a fogged-up optical lens
**Cell Injection (Supplementary Video S1)**
⬜ Monitor the abdomen to localize the tip of the scope with transillumination and to avoid over-inflation
⬜ Examine the colonic mucosa carefully to ensure its health
⬜ Avoid injection into or near a blood vessel which would lead to direct intravascular dissemination of cells and hemorrhage
⬜ Choose a suitable injection position, lift the sheath a little bit to expose the needle in the camera view⬜ Adjust the needle such that the beveled surface faces the lumen, then lower the sheath so that the needle is almost parallel to the colonic wall
⬜ Gently penetrate the colonic mucosa with the needle ensuring that its beveled edge is always facing the lumen before slowly injecting 50μL (or less) of tumor cell suspension
⬜ The first injection should be done in a more proximal location as this allows for additional injections (if desired) in increasingly distal locations with up to four possible injections per mouse
⬜ A characteristic lifting sign (Supplementary Video S1) of the mucosa during injection indicates successful injection⬜ Two investigators are optimal for the injection procedure (one navigating the endoscope, one the injection maneuver)
**After Injection**
⬜ Withdraw the needle 10 s after injection to make sure that all cells are injected
⬜ Withdraw the needle to make sure it is totally inside the sheath, then withdraw the sheath
⬜ Disinfect the endoscope, needle, and catheter using gigasept^®^ AF forte (2% *v*/*v*) and then rinse well with water
**Follow-up Colonoscopy**
⬜ Carefully examine the perianal area of the animal to ensure that there are no lesions
⬜ Gently clean the colon if feces obstruct the view and slow down air inflation speed, since tumor-burdened colorectum is fragile and easy to perforate
⬜ Keep a record of the appearance of the colonic mucosa
⬜ Score the tumor (endoscopic score) using pictures taken from colonoscopy, do not score during colonoscopy to avoid excessive air inflation⬜ Usually, one investigator is sufficient to carry out follow-up colonoscopy
**Troubleshooting, Pitfalls and General tips**
⬜ Mice should be no younger than 10 weeks to enable introduction of the endoscope without harming the animals⬜ Attach a suitable catheter to the injection needle (31G or smaller) for insertion into the working channel of the endoscope: the catheter should be rotatable within the channel to facilitate orientation of the bevel before injection (see above). On the other hand, the catheter should be thick enough so that the needle is sufficiently stabilized during injection.
⬜ If feces obstruct the view, they can usually be moved orally with gentle air inflation; if this does not work, apply 1–2 mL warm PBS to wash the colon using a soft transfer pipette
⬜ Application of more than 2 mL of PBS solution leads to a blurry/bubble-filled view of the colon and a higher risk of perforation
⬜ The use of tap water for colorectal rinsing should be avoided, since the mucosa will become less transparent or even white (bad view)
⬜ If feces or blood is coating the optical lens, withdraw the endoscope, clean it with warm PBS, and reintroduce it
⬜ If the mucosa gets folded due to peristalsis, wait a few seconds until the peristaltic wave has passed. Do not compensate by increasing air flow
⬜ Keep the mouse on a heated pad from the start of anesthesia until the mouse is fully recovered
⬜ Do not advance the endoscope anymore once you see the colonic curve (splenic flexure), maximal insertion length is approximately up to 4 cm
⬜ Limit air inflation time as increased time could cause respiratory distress, pneumoperitoneum, or even death of the mouse

## Supplementary Materials

The following are available online at https://www.mdpi.com/2072-6694/12/10/3007/s1, Video S1: Submucosal endoscopy-guided tumor cell implantation: A flexible injection catheter with a 31 G needle introduced through the working channel of the endoscope gently penetrates the mucosa in an approximately 20 to 30° angle. Subsequently, 50 μL of tumor cell suspension is injected and the characteristic lifting sign appears.
